# Plasma Inflammatory Markers and Ventriculostomy-Related Infection in Patients With Hemorrhagic Stroke: A Retrospective and Descriptive Study

**DOI:** 10.3389/fneur.2022.861435

**Published:** 2022-04-25

**Authors:** Stefan Yu Bögli, Sophie S. Wang, Elisabeth Pietrzko, Achim Müller, Amanda Eisele, Emanuela Keller, Giovanna Brandi

**Affiliations:** ^1^Neurocritical Care Unit, Department of Neurosurgery and Institute for Intensive Care Medicine, University Hospital Zürich, Zürich, Switzerland; ^2^Department of Neurology, University Hospital Zürich, Zürich, Switzerland; ^3^Clinical Neuroscience Center, University Hospital and University of Zürich, Zürich, Switzerland; ^4^Department of Neurosurgery and Neurotechnology, Eberhard Karls University Tübingen, Tübingen, Germany

**Keywords:** ventriculostomy-related infection, external ventricular drain, hemorrhagic stroke, laboratory markers, diagnostic test

## Abstract

**Background:**

Diagnosis of ventriculostomy-related infection (VRI) remains difficult due to the various existing definitions. In patients with hemorrhagic stroke, its diagnosis might be further complicated by the presence of intraventricular blood. Furthermore, hemorrhagic stroke *per se* may cause symptoms compatible with VRI. This study aimed to evaluate the benefit of plasma inflammatory markers for the diagnosis of VRI and its differentiation from patients with non-cerebral infection and patients without infection in a cohort of patients with hemorrhagic stroke.

**Methods:**

A total of 329 patients with hemorrhagic stroke and an external ventricular drain (EVD) *in situ* were admitted to the Neurocritical Care Unit, University Hospital Zurich over a period of 6 years. Of those patients, 187 with subarachnoid hemorrhage and 76 with spontaneous intracerebral hemorrhage were included. Patients with VRI were compared to patients without any infection and to patients with non-cerebral infection, with regards to their clinical characteristics, as well as their inflammatory plasma and cerebrospinal fluid (CSF) markers. For the analysis, peak values were considered.

**Results:**

The VRI was diagnosed in 36% of patients with subarachnoid and in 17% of patients with intracerebral hemorrhage. The VRI was diagnosed on an average day 9±6.2 after EVD insertion, one day after the white blood cell count (WBC) peaked in CSF (8 ± 6.3). Plasma inflammatory markers (WBC, C-reactive protein “CRP” and procalcitonin “PCT”) did not differ among patients with VRI compared to patients without infection. The CRP and PCT, however, were higher in patients with non-cerebral infection than in patients with VRI. The WBC in CSF was generally higher in patients with VRI compared to both patients without any infection and patients with non-cerebral infection.

**Conclusions:**

No differences in plasma inflammatory markers could be found between patients with VRI and patients without any infection. Conversely, CRP/PCT were higher in patients with non-cerebral infection than in patients with VRI. Altogether, CRP, PCT, and WBC are not suitable parameters for VRI diagnosis in neurocritical care unit patients.

## Introduction

External ventricular drains (EVD, ventriculostomy catheters) are used to monitor the intracranial pressure (ICP) and to drain CSF. They are particularly helpful for the management of patients with elevated ICP, secondary to acute hydrocephalus caused by subarachnoid hemorrhage (SAH) or intracerebral hemorrhage (ICH) (1). Compared to other methods of ICP monitoring, however, EVDs are associated with an increased risk of ventriculostomy-related infection (VRI)(2). Several risk factors for VRI have been identified. These include the presence of intraventricular hemorrhage, SAH, duration of catheterization, basilar cranial fracture with CSF leak, and EVD irrigation (3). The VRI is a serious complication, which has been associated with an increased rate of morbidity, mortality, length of hospital stay, and health care costs (3–5). The reported incidence of VRI varies largely from 2 up to 27% (6–10), mostly depending on the different criteria used for its diagnosis. Due to CSF contamination with blood, CSF pleocytosis, glucose, (11) and protein levels need to be carefully interpreted for the diagnosis of VRI.

Previous studies have tried to tackle the issue of the benefit of laboratory values for VRI diagnosis. Most commonly, CSF's WBC was found to be sensitive for the diagnosis of VRI (8, 12). In certain cases, however, even CSF's WBC was found to be unable to distinguish patients with and without positive CSF cultures (11). On the topic of plasma-derived markers, a recent review found that only around 30% of studies found differences in WBC within plasma, as well as C-reactive protein (CRP) between patients with and without VRI (12), with some more positive reports for the use of procalcitonin (PCT) (13, 14). Most studies, however, primarily compared patients with and without VRI excluding those with infections of another origin.

In this study, we aimed to characterize the value of CSF and plasma-derived inflammatory markers for the diagnosis of VRI in patients with hemorrhagic stroke (either non-traumatic SAH or spontaneous ICH) by comparing patients with VRI, patients with non-cerebral infection, and patients without any infection.

## Methods

The study was approved by the local ethics committee (Kantonale Ethikkommission Zürich, BASEC-Number: PB_2017_00093) and was in accordance with the ethical standards laid down in the 2013 Declaration of Helsinki for research involving human subjects.

### Study Population, Inclusion and Exclusion Criteria

This study was designed as a retrospective descriptive study. Patients were extracted from a database of consecutive adult (>18 years) patients admitted to the Neurocritical Care Unit (University Hospital of Zurich) due to hemorrhagic stroke between 2013 and 2019. Inclusion criteria were: 1) diagnosis of non-traumatic SAH or ICH, 2) EVD *in situ* for more than 48 h, and 3) at least one EVD CSF sample. Exclusion criteria were: 1) patient's written or documented oral refusal to have his/her data analyzed for research projects; 2) patients with CSF contamination (defined by positive microbiological CSF culture with skin pathogens (e.g., Staphylococcus epidermidis) without clinical or laboratory-derived suspicion of VRI).

At our institution, VRI is defined by the presence of at least two of the following conditions at least 2 or more days after insertion of EVD: a. clinical signs of VRI (fever—defined as a temperature of ≥ 38.3°C measured in-ear/bladder or >38°C measured within the brain tissue, meningeal irritability, and/or unclear neurological deterioration) not better explained by an alternative origin (e.g., increasing intracranial pressure, progression of hemorrhaging, etc.); b. WBC in CSF >500/μL; c. positive microbiological CSF culture; d. elevated systemic inflammatory parameters (CRP > 5 mg/l, PCT >.1 μg/l, and or WBC > 9.6 G/l—measured daily) not better explained by an alternative origin (excluded by performing blood, bronchial and urinary cultures, plain chest X-rays, and physical examination).

The EVDs were inserted by a neurosurgical consultant or resident in the operating room or at the intensive care unit (ICU) under sterile conditions. Prophylactic antibiotic therapy with cefuroxime was administered as a single shot based on our institutional protocol. At our center, Silver impregnated lines (Silverline^®^, Spiegelberg, Germany) are used. A CSF sampling for VRI screening is performed by an intensive care resident under sterile conditions twice per week, and, additionally, in case of VRI suspicion. After discarding the first 2 ml of CFS, 3 ml are withdrawn from the EVD for laboratory investigation. Due to the large quantities of patients with intraventricular blood (i.e., ventricular extension of the hemorrhage), CSF glucose, lactate, and protein are not routinely evaluated. The WBC within CSF is corrected for the number of erythrocytes found within the sample. In the case of VRI, empirical antibiotic therapy with intravenous vancomycin and meropenem is started and eventually modified based on the identified pathogens and their antibiotic resistance spectrum.

Clinical parameters were analyzed divided into groups with either SAH or ICH, as well as with VRI or without VRI. Laboratory parameters were analyzed between groups with SAH and ICH, as well as between groups without any infection (no VRI and no other infection), with non-cerebral infection (defined by at least one positive microbiological culture of biological material, as well as clinical signs of infection), and VRI.

### Data Collection

Patient demographics, treatment characteristics, and comorbidities, as assessed with the Charlson Comorbidities Index, antibiotic treatment, length of hospital stay, and microbiological results in CSF and plasma were collected. Additionally, systemic inflammatory parameters (CRP, PCT, and WBC), and WBC (including its microscopic differentiation) in CSF were collected. The peak values (found with EVD *in situ*) were considered for the analysis.

### Statistical Analysis

Statistical analysis was performed using SPSS version 25. Significance was defined as the probability of a two-sided type 1 error being <5% (*p-*value <.05). Descriptive statistics are reported as counts/percentages, mean ± standard deviation (SD), or as median including the interquartile range as appropriate. All continuous data were tested for normality using Shapiro–Wilk's test. Categorical variables are compared with Pearson's χ2 or Fisher's exact test, continuous/ordinal variables using Student's *t*-tests or Mann–Whitney U tests for parametric and non-parametric data, respectively, where appropriate. For comparison between multiple groups, a Kruskal-Wallis or Pearson's χ2 test, including Bonferroni correction, was used.

## Results

Of 329 patients with hemorrhagic stroke and an EVD *in situ* admitted to the NCCU of the University Hospital Zurich, 263 patients met the inclusion criteria. One hundred eighty-seven patients (71.1%) suffered from SAH and 76 patients (28.9%) from ICH. Of these patients, 36% (*n* = 68) with SAH, as well as 17% (*n* = 13) with ICH, were diagnosed with VRI. Patients' demographics and severity of disease comparing the cohorts with no infection, with non-cerebral infection, and with VRI for the different hemorrhagic stroke types are shown in Table 1. The duration of catheterization was longer in patients with VRI (18 ± 14.7) than in patients with non-cerebral infection (16 ± 12.4) or without infection (11 ± 7.4). Furthermore, the neurocritical care unit and hospital length of stay were longer in patients with VRI than the other cohorts. The severity of disease (radiographic and clinical), as well as proportion with the entricular extension of the hemorrhage, did not differ between the three cohorts.

**Table 1:** Patients' demographics and characteristics.

		**No infection**	**Non-Cerebral infection**	**VRI**
**ALL**		***n*** **= 85**	***n*** **= 97**	***n*** **= 81**
Age [years]		60 ± 14.9	61 ± 12.3	56 ± 12.9
Gender (Male)		39 (45.9)	45 (46.4)	36 (44.4)
Charlson comorbidity index		0 [0, 1.5]	0 [0, 1]	0 [0, 1]
Type of hemorrhage *^a^*	SAH	52 (61.2)	67 (69.1)	68 (84.0)
	ICH	33 (38.8)	30 (30.9)	13 (16.0)
Initial GCS ≤ 8		35 (41.2)	53 (54.6)	37 (45.7)
Ventricular extension		48 (56.5)	60 (61.9)	46 (56.8)
EVD days *in situ* [d] *^a^, ^b^, ^c^*		11 ± 7.4	16 ± 12.4	18 ± 14.7
Antibiotic treatment *^a^, ^c^*		49 (57.6)	96 (99.0)	56 (69.1)
NCCU length of stay [d] *^a^, ^b^, ^c^*		17 ± 11.3	23 ± 11.5	24 ± 13.0
Hospital length of stay [d] *^b^*		25 ± 13.9	30 ± 16.3	33 ± 17.4
**SAH**		***n*** **=** **52**	***n*** **=** **67**	***n*** **=** **68**
Age [years]		60 ± 14.1	61 ± 12.4	57 ± 13.3
Gender (Male)		20 (38.5)	24 (35.8)	27 (39.7)
Charlson comorbidity index		0 [0, 1.75]	0 [0, 1]	0 [0, 1]
Fisher grade	1	1 (1.9)	0 (0.0)	1 (1.5)
	2	2 (3.8)	0 (0.0)	3 (4.4)
	3	21 (40.4)	27 (40.3)	32 (47.1)
	4	28 (53.8)	36 (53.7)	32 (47.1)
WFNS grade	1	4 (7.7)	10 (14.9)	21 (30.9)
	2	14 (26.9)	10 (14.9)	10 (14.7)
	3	2 (3.8)	4 (6.0)	2 (2.9)
	4	17 (32.7)	17 (25.4)	18 (26.5)
	5	15 (28.8)	22 (32.8)	17 (25.0)
Ventricular extension		22 (42.3)	35 (52.2)	35 (51.5)
EVD days *in situ* [d] *^a^*		13 ± 7.8	17 ± 7.3	18 ± 15.4
Antibiotic treatment *^a^, ^c^*		28 (53.8)	67 (100)	47 (69.1)
NCCU length of stay [d]		20 ± 12.1	24 ± 12.0	24 ± 13.1
Hospital length of stay [d]		27 ± 13.9	32 ± 17.7	32 ± 17.1
**ICH**		***n*** **=** **33**	***n*** **=** **30**	***n*** **=** **13**
Age [years]		61 ± 16.2	58 ± 11.9	53 ± 10.9
Gender (Male)		19 (57.6)	21 (70.0)	9 (69.2)
Charlson comorbidity index		0 [0, 1.5]	0 [0, 1]	0 [0, 1.5]
Initial GCS ≤ 8		15 (45.5)	18 (60.0)	9 (70)
Ventricular extension		26 (78.8)	25 (83.3)	11 (84.6)
EVD days *in situ* [d] *^a^, ^b^*		8 ± 5.7	16 ± 19.7	15 ± 10.1
Antibiotic treatment *^a^, ^c^*		21 (63.6)	29 (96.7)	9 (69.2)
NCCU length of stay [d] *^a^*		22 ± 13.8	24 ± 10.9	35 ± 19.1
Hospital length of stay [d]		22 ± 13.8	24 ± 10.9	34 ± 19.1

a *p-value is significant for no infection vs. non-cerebral infection,*

b *p-value is significant for no infection vs. VRI,*

c *p-value is significant for non-cerebral infection vs. VRI, VRI ventriculostomy-related infection; SAH, subarachnoid hemorrhage; ICH, intracerebral hemorrhage; EVD, external ventricular drain; GCS, Glasgow Coma Scale; NCCU, neurocritical care unit*.

**Figure 1:**
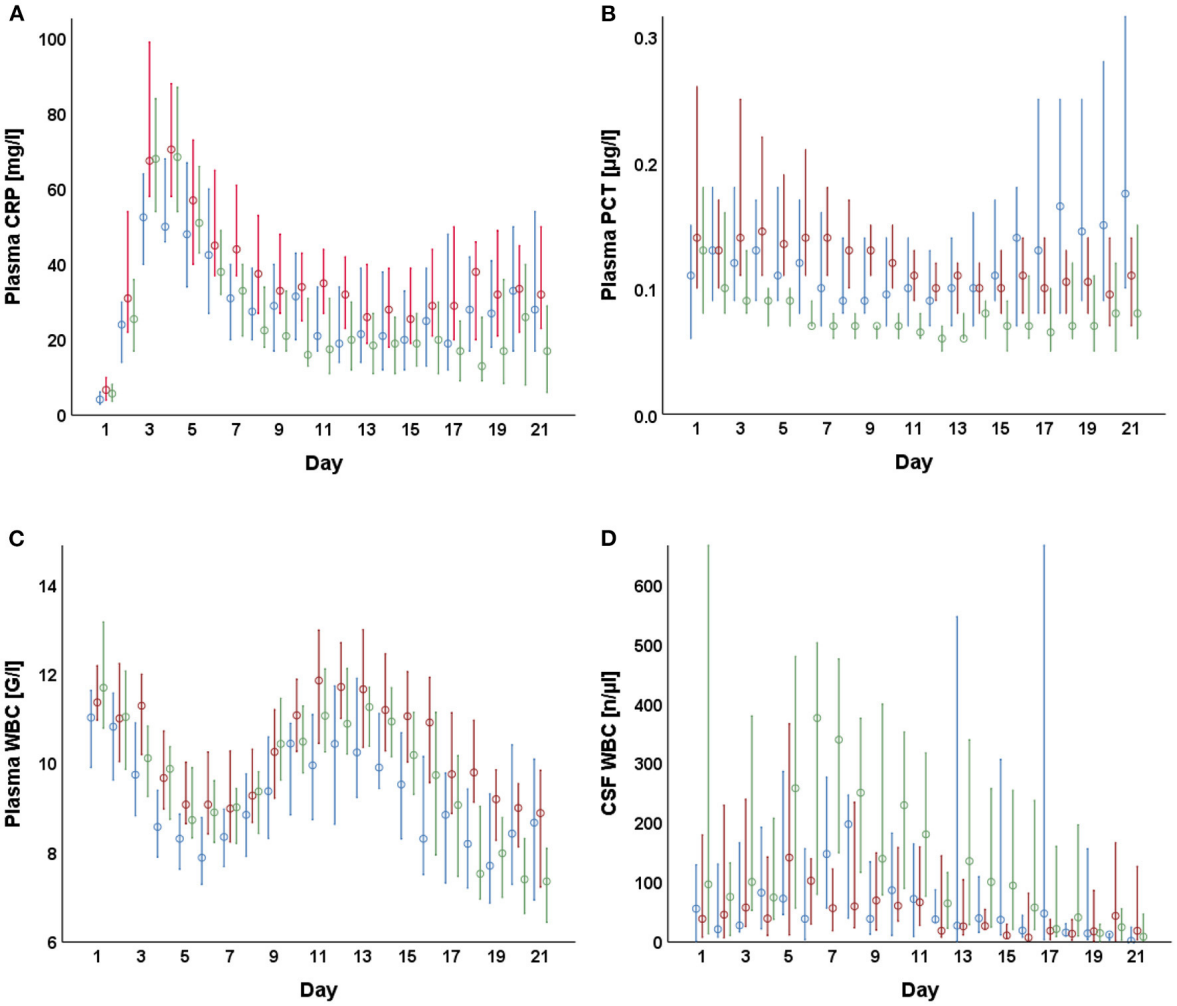
Plasma and cerebrospinal fluid (CSF)-derived parameters after external ventricular drain (EVD) insertion. Median daily values including the 95% confidence interval between day 1 and 21 are shown for CRP **(A)**, PCT **(B)**, plasma WBC **(C)**, as well as CSF WBC **(D)** grouped by control (blue), control with non-cerebral infection (red), and VRI (green).

**Figure 2:**
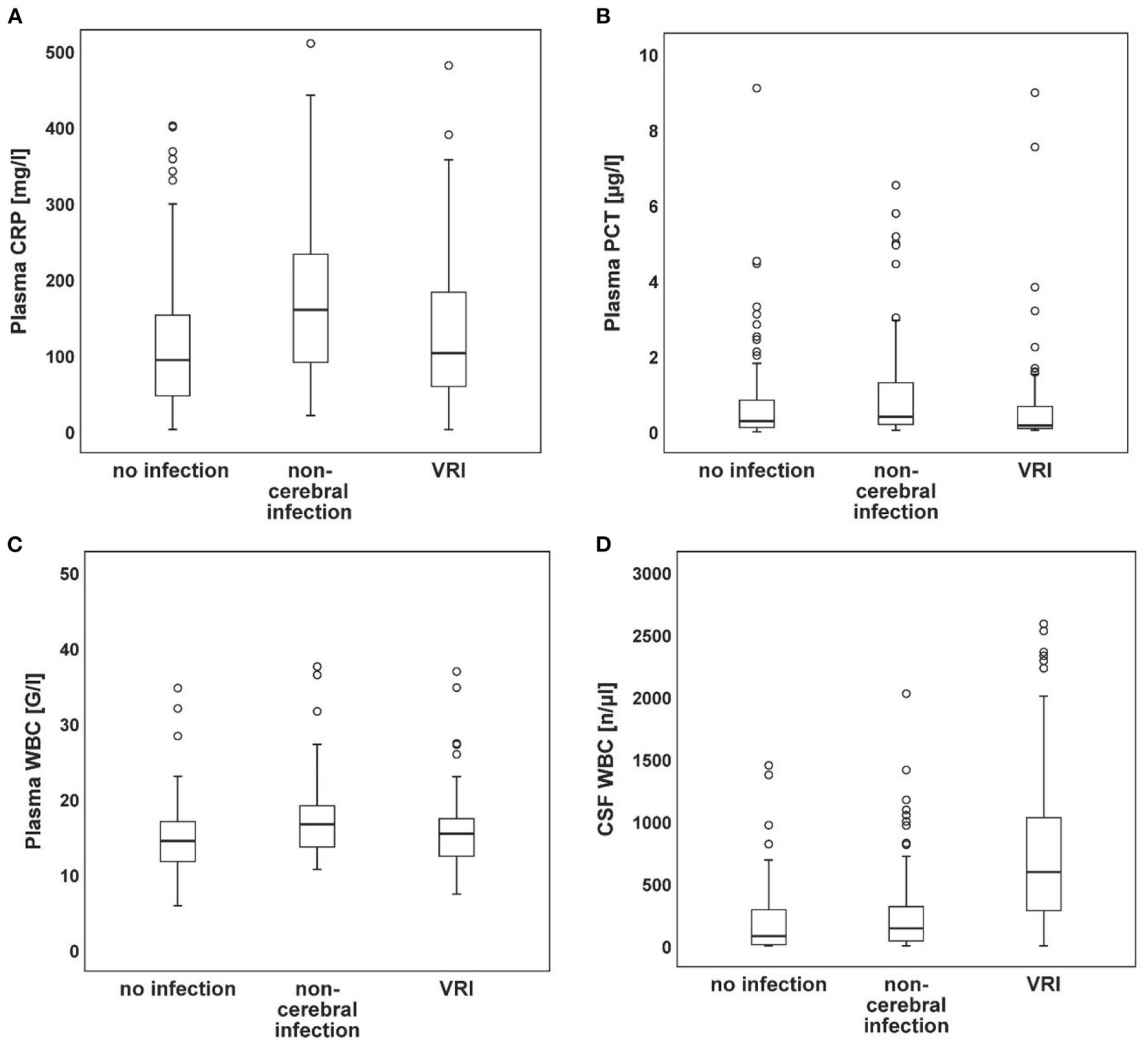
Peak values of plasma and CSF-derived parameters. Box plots of CRP **(A)**, PCT **(B)**, plasma WBC **(C)**, and CSF WBC **(D)** peak values showing the median (horizontal line), the first and third quartile (lower and upper end of box), the minimum and maximum (whiskers), and outliers (circles; values outside 1.5 times the interquartile range).

The laboratory parameters of CSF and plasma are presented in Table 2. The course of WBC in CSF and plasma, as well as of CRP and PCT, are shown in Figure 1. Figure 2 shows the distributions at their respective peak. Overall, patients with non-cerebral infection had significantly higher inflammatory markers in plasma compared to patients without any infection. Similarly, patients with non-cerebral infection had higher CRP and PCT than patients with VRI. No differences in plasma inflammatory markers could be found between patients with VRI and patients without any infection. The WBC in CSF was higher in patients with VRI compared both to other groups. Considering patients with SAH and ICH separately, WBC in plasma did not differ in patients with SAH among patients with VRI, with non-cerebral infection, and without any infection. Overall, WBC in plasma and CSF was higher in patients with VRI following SAH than in patients with VRI following ICH.

**Table 2:** Laboratory parameters.

**All patients (SAH and ICH)**	**No infection** **(0)**	**Infection, noVRI** **(1)**	**VRI** **(2)**	***p*-value**	***p*** **-value of subgroup-comparison**		
	***n* = 85**	***n* = 97**	***n* = 81**	**KW-test**	**0 vs. 1**	**0 vs. 2**	**1 vs. 2**
**Plasma CRP**	**116 ± 92.1**	**176 ± 106.1**	**134 ± 96.5**	** <0.001**	** <0.001**	**0.525**	**0.013**
**Plasma PCT**	**1.3 ± 3.32**	**5.7 ± 23.52**	**2.6 ± 12.03**	**0.002**	**0.076**	**0.667**	**0.002**
**Plasma WBC**	**15 ± 4.8**	**24 ± 45.8**	**16 ± 5.1**	**0.001**	** <0.001**	**0.537**	**0.059**
**CSF WBC**	**362 ± 1205.4**	**514 ± 1562.3**	**2041 ± 10250.8**	** <0.001**	**0.418**	** <0.001**	** <0.001**
**SAH**	**No infection (0)**	**Infection, noVRI** **(1)**	**VRI (2)**	***p*-value**	***p*** **-value of subgroup-comparison**		
	***n* = 52**	***n* = 67**	***n* = 68**	**KW-test**	**0 vs. 1**	**0 vs. 2**	**1 vs. 2**
**Plasma CRP**	**116 ± 90.1**	**171 ± 104.2**	**140 ± 99.9**	**0.005**	**0.004**	**0.524**	**0.137**
**Plasma PCT**	**1.1 ± 2.23**	**2.0 ± 4.63**	**3.0 ± 13.09**	**0.035**	**0.600**	**0.770**	**0.029**
**Plasma WBC**	**16 ± 5.2**	**20.9 ± 34.7**	**16 ± 5.4**	**0.180**			
**CSF WBC**	**336 ± 700.8**	**685 ± 1855.9**	**2174 ± 11096.5**	** <0.001**	**1.000**	** <0.001**	** <0.001**
**ICH**	**No infection (0)**	**Infection, noVRI** **(1)**	**VRI (2)**	***p*-value**	***p*** **-value of subgroup-comparison**		
	***n* = 33**	***n* = 30**	***n* = 13**	**KW-test**	**0 vs. 1**	**0 vs. 2**	**1 vs. 2**
**Plasma CRP**	**115 ± 96.7**	**189 ± 111.0**	**107 ± 73.6**	**0.008**	**0.013**	**1.000**	**0.077**
**Plasma PCT**	**1.7 ± 4.57**	**13.9 ± 41.01**	**1.0 ± 2.07**	**0.037**	**0.107**	**1.000**	**0.084**
**Plasma WBC**	**13 ± 3.7**	**30 ± 63.8**	**13 ± 2.2**	**0.001**	**0.001**	**1.000**	**0.012**
**CSF WBC**	**403 ± 1740.3**	**134 ± 155.8**	**1348 ± 3522.6**	**0.006**	**0.416**	**0.004**	**0.135**
**SAH vs. ICH**	**No infection**	**Infection, no VRI**	**VRI**				
**MWU-test**	***p*-value**	***p*-value**	***p*-value**				
**Plasma CRP**	**0.735**	**0.492**	**0.368**				
**Plasma PCT**	**0.749**	**0.200**	**0.657**				
**Plasma WBC**	**0.031**	**0.410**	**0.016**				
**CSF WBC**	**0.001**	**0.010**	**0.052**				

*Statistics are performed using either a Kruskall Wallis test or a Mann-Whitney- U test, subgroup p-values are shown adjusted for multiple comparison using Bonferroni correction. CRP is described in mg/l, PCT in μg/l, WBC in G/l, and CSF WBC in n/μl. VRI, ventriculostomy related infection; SAH, subarachnoid hemorrhage; ICH, intracerebral hemorrhage; CRP, C-reactive protein; PCT, procalcitonin; WBC, white blood cell count; CSF, cerebrospinal fluid; KW-test, Kruskal Wallis test; MWU-test, Mann-Whitney-U test*.

Considering patients with VRI (Table 3), a VRI was diagnosed on average on day 9 ± 6.2 after EVD insertion. The time duration between EVD insertion and VRI diagnosis did not differ among patients with SAH and ICH (8 ± 5.5 vs. 11 ± 8.9, *p* = 0.114). Furthermore, the WBC peak in CSF occurred at similar time points in SAH and ICH (8 ± 6.3 vs. 7±6.3 for SAH and ICH respectively) (*p* = 0.874). Neutrophils were the most frequent subtype of WBC at the day of the peak in both patients with SAH (55 ± 25.5%) and ICH (59 ± 32.1%). Fourteen (17.2%) patients presented positive CSF culture (N = 3 Escherichia coli, N = 3 Klebsiella pneumoniae; N = 1 Enterococcus faecalis; N = 1 Enterococcus cloacae; N = 1 Klebsiella aerogenes; N = 1 Pseudomonas aeruginosa; N = 1 Serratia marcescens; N = 2 Staphylococcus epidermidis; and N = 1 Streptococcus pyogenes). No differences in CSF or plasma-derived factors, as well as the timing of peak and date of diagnosis, could be found comparing patients diagnosed with VRI, either with or without a positive CSF culture.

**Table 3:** Subgroup analysis of patients with ventriculostomy-related infection (VRI).

**VRI CSF parameters and characteristics**		**All** ***n* = 81**	**SAH** ***n* = 68**	**ICH** ***n* = 13**	***p*-value**	
Day VRI diagnosis		9 ± 6.2	8 ± 5.5	11 ± 8.9	0.114
Peak CSF WBC day		8 ± 6.3	8 ± 6.3	7 ± 6.3	0.874
Peak CSF WBC		2063 ± 10313.4	2174 ± 11096.5	1348 ± 3522.6	0.792
Neutrophils		55 ± 26.3	55 ± 25.5	59 ± 32.1	0.656
Lymphocites		27 ± 20.8	27 ± 19.6	28 ± 28.2	0.850
Macrophages/Monocytes		15 ± 8.7	16 ± 7.9	12 ± 12.5	0.270
Eosinphiles		2 ± 7.7	2 ± 8.4	0 ± 0.3	0.638
Basophiles		0 ± 0.5	0 ± 0.4	0 ± 0.8	0.789
Plasmaells		0 ± 0.2	0 ± 0.2	0 ± 0.0	0.355
**VRI with and without positive CSF culture**	**VRI without positive culture**	**VRI with positive culture**	***p*** **-value**
	***n*** **=** **67**	***n*** **=** **14**	
Plasma CRP	127 ± 89.4	174 ± 121.7	0.110
Plasma PCT	3.1 ± 13.19	0.5 ± 0.66	0.499
Plasma WBC	15 ± 4.5	19 ± 7.0	0.078
CSF WBC	742 ± 652.3	8263 ± 24373.8	0.181
Day of VRI Dig	8 ± 5.8	12 ± 7.5	0.137
Peak WBC day	7 ± 5.4	11 ± 8.8	0.232
Peak WBC	748 ± 652.2	8263 ± 24373.8	0.314
Neutrophils	54 ± 24.4	60 ± 32.4	0.476
Lymphocites	27 ± 18.2	28 ± 28.4	0.576
Macrophages/Monocytes	16 ± 7.5	12 ± 11.8	0.094
Eosinphiles	2 ± 8.9	0 ± 0.6	0.919
Basophiles	0 ± 0.5	0 ± 0.2	0.416
Plasmaells	0 ± 0.2	0 ± 0.0	0.492

*Statistics are performed using a t-test or a Mann-Whitney- U test. CRP is described in mg/l, PCT in μg/l, WBC in G/l, and CSF WBC in n/μl. VRI, ventriculostomy related infection; SAH, subarachnoid hemorrhage; ICH, intracerebral hemorrhage; CRP, C-reactive protein; PCT, procalcitonin; WBC, white blood cell count; CSF, cerebrospinal fluid*.

Patients with non-cerebral infection most commonly suffered from pneumonia (*n* = 76, 78%), followed by urinary tract infections (*n* = 12, 12%), with another 4 (4%), 3 (3%), and 2 (2%) suffering from gastrointestinal, intravenous catheter-related, and other infections, respectively; Furthermore, patients without infection commonly presented with fever (*n* = 25, 29.4%) or neurological deterioration during their stay (*n* = 29, 34.1%).

## Discussion

The purpose of our study was to evaluate the benefit of the plasma inflammatory markers WBC, CRP, and PCT for the diagnosis of VRI and its differentiation from patients with non-cerebral infection and patients without infection. While patients with infection of non-cerebral origin had higher systemic inflammatory markers within plasma than patients with VRI, there were no significant differences in levels of CRP, PCT, or plasma WBC between patients with VRI and controls without infections.

The finding that plasma-derived laboratory values do not differentiate patients with VRI and controls without infections is of interest. However, this study has several limitations. Firstly, this is a single-center retrospective study limiting the generalizability of the results. Furthermore, the true incidence of VRI is unknown. At our institution, VRI is diagnosed by the presence of two or more signs/symptoms of VRI after exclusion of alternative infectious source, and earliest 48 h after EVD insertion. This definition follows the criteria by the Centers for Disease Control and Prevention (CDC) (15). Due to the retrospective design of this study, the exact combination of symptoms and signs are unknown. Positive CSF culture was not necessary. Thus, some patients without VRI might have been wrongly grouped. While this definition allows for early diagnosis, it comes with its benefits and drawbacks that we describe below.

The VRI currently lacks universally accepted reliable diagnostic criteria with over 16 unique definitions reported (16). The reported frequency of VRI ranges between 2% and 27%. Simple definitions rely purely on one or more positive CSF cultures with or without concomitant change within the CSF laboratory values (6–10). Due to the high mortality and morbidity associated with VRI (3–5), antibiotic therapy is (also in our cohort) commonly started empirically in the absence of positive bacterial CSF cultures, as the achievement of negative results from cultures requires at least 72 h (17, 18). On the other hand, even though CSF culture remains the gold standard, false-negative tests occur in up to 20% (12, 19). Patients with positive PCR and CSF-negative cultures have been described, suggesting some “aseptic” infections to have a bacterial origin (19). Increasing the frequency of CSF sampling is also not a viable solution as sampling itself increases the positive rate of cultures (20, 21).

More lenient criteria only require clinical signs of VRI, as well as a plasma or CSF-derived laboratory values, in different combinations. Hemorrhagic stroke *per se* may cause headache, impaired level of consciousness, fever, and nuchal stiffness leading to a difficult interpretation of these clinical parameters (22). Furthermore, blood contamination of CSF hampers the interpretation of cell count, glucose, protein, and lactate levels (23). The SAH of aneurysmal origin itself causes a systemic inflammatory response that includes an increase of WBC, CRP, and PCT within plasma in the absence of a microbial origin (24–26).

## Conclusion

The rise of plasma-derived laboratory markers allows for the detection of an infection. However, in our cohort of patients with hemorrhagic stroke, CRP, PCT, and WBC did not prove to be reliable parameters for the diagnosis of VRI. In case of increased inflammatory markers in plasma, a non-cerebral infection is more likely. In the setting of an ICU, patients are critically ill and have several invasive devices and lines (e.g., endotracheal arterial catheters, central venous, and arterial lines) and are, thus, more prone to associated infections (bloodstream infection, pneumonia, or urinary tract infection), which affect the systemic inflammatory response. Furthermore, SAH itself leads to a systemic inflammatory response rendering this patient cohort at risk of overdiagnosing and overtreatment in comparison to ICH. Better diagnostic criteria for VRI are, thus, needed in this specific patient group.

## Data Availability Statement

The raw data supporting the conclusions of this article will be made available by the authors, without undue reservation.

## Ethics Statement

The studies involving human participants were reviewed and approved by Kantonale Ethikkommission Zürich, BASEC-Number: PB_2017_00093. Written informed consent for participation was not required for this study in accordance with the national legislation and the institutional requirements.

## Author Contributions

SB, SW, and GB conceived the study. EP, AM, and AE acquired some of the data. Statistical analysis was performed by SB. SB and GB drafted the manuscript. EK reviewed the manuscript for intellectual content. All authors agreed with the publication and the manuscript.

## Conflict of Interest

The authors declare that the research was conducted in the absence of any commercial or financial relationships that could be construed as a potential conflict of interest.

## Publisher's Note

All claims expressed in this article are solely those of the authors and do not necessarily represent those of their affiliated organizations, or those of the publisher, the editors and the reviewers. Any product that may be evaluated in this article, or claim that may be made by its manufacturer, is not guaranteed or endorsed by the publisher.
